# “I can’t have it; I am a man. A young man!” – men, fibromyalgia and masculinity in a Nordic context

**DOI:** 10.1080/17482631.2019.1676974

**Published:** 2019-10-13

**Authors:** Merja Sallinen, Anne Marit Mengshoel, Kari Nyheim Solbrække

**Affiliations:** a Faculty of Health and Wellbeing, Satakunta University of Applied Sciences, Pori, Finland; b Institute of Health and Society, University of Oslo, Oslo, Norway; c Department of interdisciplinary health sciences, Institute of Health and Society, University of Oslo, Oslo, Norway

**Keywords:** Nordic countries, narrative research, life-story interview, chronic pain, gender

## Abstract

**Purpose**: Research shows that gender has a substantial impact on the health behaviour such as expression of physical symptoms like persistent pains and aches. However, there is yet little knowledge about the gendered aspect of pain by men who suffer from typical female diseases like fibromyalgia. The purpose of the study was to elucidate the interplay between illness and gender by exploring life-stories of men who suffer from fibromyalgia.

**Methods**: The data were collected through life-story interviews of eight men suffering from fibromyalgia. A narrative methodology for analysis was applied to explore the storytelling and the linguistic and performative aspects of the life-stories.

**Results**: The masculine identity of the participants was re-negotiated by comparisons to other men and life before symptom onset, and by discussing expectations and beliefs of how men should act in contemporary societies. The transition from experiencing a strong, active and reliable body to experiencing a painful, vulnerable and helpless body was perceived as fundamental.

**Conclusions**: Self-management and rehabilitation of fibromyalgia it is not only about learning to manage the symptoms but also about the struggle to find coherence in life through re-constructing gender identity that is acceptable both for the individual and for the community.

## Introduction

The onset and development of chronic widespread pain can be a threatening experience to one’s gender identity. Research shows that gender has a significant impact on health behaviour such as help seeking or on expression of physical symptoms (Boerner et al., 2018; Ek, 2013; Flurey et al., 2017; O’Brien, Hart, & Hunt, 2007; O’Brien, Hunt, & Hart, 2005; O’Loughlin et al., 2011). Concerning chronic pain, research shows that men tend to adopt a “wait and see” approach more often than women, especially if the symptoms are perceived as minor and not life threatening (Keogh, 2015; Paulson, Danielsson, & Söderberg, 2002; Paulson, Norberg, & Danielson, 2002). Hence, men might not seek for medical help for their pains and aches, but rather stand the situation “like a man” unless the symptoms start to interfere with their work or family life (Ahlsen, Bondevik, Mengshoel, & Nyheim Solbraekke, 2014; O’Brien et al., 2007, 2005; Paulson et al., 2002).

In a recent review, Boerner et al. (2018) noted that differences in pain experiences between males and females based on their biologically determined sex were well known. However, the researchers pointed out that there is yet little knowledge about social or cultural aspects of pain experience, which may indeed appear different for males and females. The presence of chronic pain does not allow individuals to achieve the most valued standards of being male or female in our societies (Bernardes & Lima, 2010). In his review on men, masculinity and pain, Keogh (2015) noticed that barriers men face around help seeking include ineffective communication skills, embarrassment and anxiety. He emphasized that a greater understanding of the reciprocal relationship between living with pain and masculinity is required.

In this article, we aim at elucidating the interplay between illness and gender by exploring life-stories of men with fibromyalgia. The general outline of the article is as follows: firstly, we introduce the concept “gender” in relation to health and illness with a special interest in chronic pain and fibromyalgia. Secondly, we address storytelling and narrative methodology as a way of illuminating the world of the ill person and describe how we collected and analysed the data in this study. Finally, we explore in-depth one particular life story, which crystallizes what might be at stake when men are being diagnosed with fibromyalgia.

## Gender, health and illness

According to West and Zimmerman (1987), the concept “gender” refers to culturally accepted norms, attitudes and beliefs of what it is to be a man or a woman. Further, they pointed out that while gender by common sense is conceived as something that an individual *is*, current understanding of gender emphasizes that it is something an individual *does* through the behaviours and interactions with others. In contrast to the more old school term “gender role”, West and Zimmerman underlined that gender is to be understood as a continuous process, rather than a fixed entity or result, and that it is linguistically constructed and contextually situated. Hence, person’s acts and behaviours are critical to their ability to fit into cultural gender norms and deviating from the norms can be met with negative stigma or censure from peers and at worst, with social exclusion (Fleming & Agnew-Brune, 2015).

Taking this doing-gender perspective as a general point of departure, the concept *hegemonic masculinity* (introduced by Raewyn Connell 1987) is widely used to describe the Western ideal of a “true man”. Hegemonic masculinity is a cultural ideal characterized by power and dominance (over women) as well as by strength, fortitude, endurance, and independence, which presuppose the presence of a healthy body (Branney & White, 2008; Jewkes et al., 2015). Consequently, qualities like expressiveness, bodily weakness, caring attitude and dependence on others are often associated with femininity (Bernardes & Lima, 2010; Keogh, 2015). However, the concept of hegemonic masculinity portraits an ultimate ideal that can be achieved only by western, white, healthy, heterosexual men in societal positions characterized with authority and power, if at all. Therefore, the concepts of masculinity have been developed further both by Connell and by other scholars (Connell & Messerschmidt, 2005). *In marginalized masculinity,* the person lacks some characteristics of hegemonic masculinity (e.g., is disabled), whereas in *subordinated masculinity* one has qualities opposite to hegemonic masculinity (e.g., being overly emotional). The concept *complicit masculinity*, in turn, refers to not having all the qualities of hegemonic masculinity, but not challenging it either. Moreover, it has been emphasized that *alternative masculinities*, which were previously not considered as hegemonic are entering the fray. Men in predominantly “female” jobs (like nursing or kindergarten teaching) or active, family-oriented, involved fathers are becoming more accepted and supported in the society, and thus, the idea of what it is to be a man in the contemporary society is changing (Buschmeyer & Lengersdorf, 2016).

As Solbraekke (2016) pointed out, in the Nordic countries the gender relations and norms have changed substantially over the recent decades. That is, men are not anymore strictly expected to act in a stoic and instrumental way, and women are increasingly encouraged to act beyond the traditional feminine script of weakness and endless caring. In concordance with this, Ahlsen and Solbraekke (2017) noted that although the cultural expectations that men are physically and emotionally strong and independent was expressed in illness narratives of male pain patients, their illness stories intersected with less typically masculine aspects, such as relational disruptions and emotional distress, and expressions of need for comfort and care.

Several scholars suggest that the body is often the central foundation of how men define themselves and how others define them (Ahlsen & Solbraekke, 2017; Emslie, Ridge, Ziebland, & Hunt, 2006; O’Brien et al., 2005; Robertson, Sheikh, & Moore, 2010). However, the constitution of masculinity through bodily performances, such as sports or manual labour, means that gender is vulnerable if the performance cannot be sustained due to illness or physical disability (Connell & Messerschmidt, 2005). For example, Flurey et al. (2017) noticed that men with rheumatoid arthritis re-negotiated their masculinity in different ways and had to re-write their masculinity scripts to enable them to accept and adapt to their condition. Some of them seemed to push through the pain to retain their masculine activities (such as sports), whereas some others replaced them with other activities (Flurey et al., 2017).

O’Brien et al. (2007) remarked that many studies exploring the relation between masculinity and chronic illnesses, have examined experiences of men with gender-specific diseases such as prostate cancer, or diseases that are more common in male than in female population (e.g., coronary heart disease). For example, although the men with prostate cancer felt that their masculinity had been mutilated or “cut off”, they were ready to accept the changes because they had immediate concerns about their survival and discussion about gender identity was not a priority in their lives. The men with coronary heart disease, in turn, believed that losing the workability or their role as the breadwinner in the family was also a loss of masculine identity, at least to some extent. O’Brien et al. (2007) pinpointed that there are only a few studies focusing on illnesses that are rare in male population but common among women. However, it is stated conditions that are more common in women than in men (such as depression or fibromyalgia), may challenge the masculine identity even more than the typically male illnesses and thus, have a wider de-masculinizing effect (Bernardes & Lima, 2010; Branney & White, 2008; Emslie et al., 2006; O’Brien et al., 2007). Some studies have suggested that for instance mental health issues or chronic pain complaints are perceived as “female problems” and are not necessarily disclosed in the male patients’ encounters with health professionals colleagues due to the fear of being labelled “sissy” or “un-manly” (Bernardes & Lima, 2010; Emslie et al., 2006; O’Brien et al., 2007, 2005; Paulson et al., 2002; Samulowitz, Gremyr, Eriksen, & Hensing, 2018).

Fibromyalgia is commonly perceived as “women’s illness” because until recently 80–90% of the patients in clinical settings have been women in their mid-life (Arnold, Clauw, & McCarberg, 2011). However, as Arnold et al. (Arnold et al., 2011) pointed out, despite greater interest in and awareness of the disorder than ever before, fibromyalgia remains yet underdiagnosed and undertreated, especially among men. It is noteworthy, that fibromyalgia has one of the lowest rankings in the prestige hierarchy of illnesses together with some other female-dominated illnesses like depression or eating disorders (Album & Westin, 2008; Grue, Johannessen, & Fossan Rasmussen, 2015). In illnesses like fibromyalgia, the credibility of the patients might be questioned by the health professionals due to the lack of clear physical findings, e.g., in x-rays or blood tests: “less physical” illnesses are often conceived as being “less real” (Conrad & Barker, 2010; Grue et al., 2015; Jutel, 2011). According to Conrad and Barker (Conrad & Barker, 2010), physicians, lay public and sometimes even the fibromyalgia patients themselves question the authenticity of the symptoms and the credibility of the suffering. Katz, Mamyrova, Guzhva, and Furmark (2010) in turn, noticed that physicians were reluctant to assign a label of fibromyalgia to men because it is perceived as a “women’s illness”. In addition, Colmenares-Roa et al. (2016) pointed out that medical doctors see people with fibromyalgia as difficult patients due to long consultation times, non-improvement and poor compliance to treatment. In addition, Sallinen and Mengshoel (2017) concluded in their recent study that men with fibromyalgia seem to carry a double burden in terms of credibility: having a contested diagnosis and being a man with “women’s illness”.

However, due to the revised diagnostic criteria for fibromyalgia (ACR2010), it is evident that we will encounter an increasing number of male patients who fulfil the criteria and get fibromyalgia diagnosis (Jones et al., 2015; Wolfe, Brähler, Hinz, & Häuser, 2013). Hence, it is timely to examine the illness experiences of men with fibromyalgia. In terms of social construction of illness and gender, it is necessary to explore how do they “*do*” and negotiate gender in their life stories and how these negotiations intersect with dominant norms of masculinity.

## Narrative approach as a methodological frame

The understanding of telling narratives as a fundamental way for humans to give meaning to their own experiences is a theoretical framework underpinning this study (Davies, 2002; Hydén, 1997). According to Mattingly and Lawlor (2000), narratives are event-centred and historically particular, and located in a specific time and place. Davies (2002) emphasized that human stories can be conceived from three perspectives: firstly, as factual presentations of events, secondly, as cultural scripts that supply guidelines for understanding actions, and thirdly, as performances that re-create and comment past events. Bamberg, De Fina, and Schiffrin (2011) in turn, underlined the role of storytelling as a tool to construct and reconstruct one’s identity. They pointed out that the question is to a lesser degree about what actually happened and to a much larger degree how constancy and change in identity are constructively navigated.

By making the story “tellable”, the speaker is not only reporting but also inviting the listener to join in contemplating it, as well as in responding to it (Davies, 2002; Mattingly & Lawlor, 2000; McMahon et al., 2012; Riessman, 2003). As pointed out by Gubrium and Holstein (1998), the listeners collaborate in both the “whats” and “hows” of the story by invoking cultural meanings and expectations in relation to the local auspices of the narration. In the present study, the interviewers represented the audience for the told life stories but at the same time, they acted as facilitators for the narrative process. Hence, a narrative approach was used both as a theoretical point of departure and as a method of collecting and analysing the data which will be elaborated in the following.

## Data collection

The data were elicited from interviews of men with fibromyalgia who were invited to share their life stories with the researchers. The participants of the study were reached through Finnish and Norwegian rheumatism/fibromyalgia associations and through a Norwegian rehabilitation centre. We asked if men who were diagnosed with fibromyalgia were interested in participating in a research process and invited them to contact the researchers. Time and place for an interview was then agreed with each participant individually.

Originally, 10 men agreed to be interviewed but 2 of them withdrew from the study before the interviews were started. Thus, eight men (average age 47 years, ranging from 24 to 61 years) with different professional, educational and social backgrounds participated in the study. Six of the participants lived with a spouse or family and two of them were single. Five of them were working in full-time or part-time jobs, e.g., within engineering or marketing/sales, whereas three participants were on permanent or temporary disability benefits. The interviews took place in participants’ home, at the rehabilitation centre or at the university depending on which location was most convenient for each participant. We asked and obtained a written consent to participate from all participants before the interviews.

The interview method was inspired by a 3-stage interview technique described by Rosenthal (2003). First, the interviewer asked the participants to tell his life story in his own words. No time limit for this part was given, and the interviewer encouraged the participants to include whatever they found important and to exclude what they did not want to talk about. In the second part of the interview, the interviewer used a semi-structured interview technique asking questions that emerged from the storytelling. The purpose was to fulfil the gaps in the story and to encourage the participant to elaborate and deepen his story. In the final part of the interview, the contribution as such and the interview experience in general were discussed. In some cases, new stories evolved at this point, as the participant recalled some important events or discussions, or wanted to add on to the story on more emotional level. The interviews took in average 2 h (ranging from 1 to 3 h) and were recorded following the permission of the participant.

## Data analysis

The analysis of the narratives was inspired by the work of Mattingly and Lawlor (2000) and Riessman (2003). They suggested that the in-depth narrative analysis could focus on one-two cases that are especially evoking or revealing regarding the research questions. Riessman (2003) underlined the importance of analysing the process of telling a narrative and narrator’s strategic choices in the narrative about positioning of characters, audience, and self. Moreover, she emphasized the analysis of the performative aspects of narratives, i.e., the displays of self and identity that are not only spoken words but also enacted or embodied actions. She remarked that the displays of identity go beyond the verbal in a strict sematic sense; what is the text about or what it “says”. As Mattingly and Lawlor (2000) showed, the choice of wordings, the use the present or past tense or active or passive voice may pinpoint or nuance the important turning points in the story and give the teller a possibility to distance from her/his individual experiences.

In the present study, the interviewers (first and second author) shared the first impressions after each interview and discussed the main contents and evocative issues, which seemed to be particularly important for the given participant. The recorded data were first listened to several times to reach the “whole” of the story. The interviews were transcribed verbatim and the transcripts were checked against the recorded material. In the first stage of the analysis, the focus was on the content: *what did they tell*? *What are the commonalities in the stories?* However, as we were interested in *how* the men in question “*did*” and negotiated their gender in their accounts, we put special emphasis on analysing the rhetorical means the men used. Thus, the examples the men gave, the metaphors and phrasings they used and the overall rationale behind the life story were examined in detail. Following the ideas of Riessman (2003), we also paid attention to the subject positions the participants took and the nonverbal communication they used.

To present not only the content but also the process of narrative storytelling, we have chosen to present the result of our analyses by displaying one particular illness story, that of “Steve” (pseudonym). The story we have chosen to be presented here, is of course highly individual, which is reflecting Steve’s uniqueness as a human being. At the same time though, we have chosen it because we believe it offers an extremely vivid general manifestation on how it is to be a man diagnosed with fibromyalgia in contemporary Nordic societies. Moreover, he is young, in his mid-twenties. Hence, the age and sex of the narrator create inherent tensions between wider societal expectations, beliefs and attitudes concerning a typical fibromyalgia-patient, on the one hand, and Steve’s social situation and story on the other. During his storytelling, Steve “set the scene” for the events and performed the lines of different actors by changing his voice, and thus created a vivid picture not only of what happened but also how discussions evolved and how he felt about all that at the time of the events and now retrospectively. Moreover, he approached his life events from several different perspectives, thus producing a rich and polyphonic life story for analysis.

In the following extracts, some details have been changed or blurred to protect the privacy of the participant. Some further information is provided in square brackets [e.g., expressed emotions] to help the reader to understand the quote. Pausing in speech is denoted with three dots (…), omitted text with three dots in square brackets […] and emphasis of words by underlining.

## Ethical considerations

The present study was a part of a larger research project and was conducted in accordance with the Helsinki Declaration. The local ethical committees in both Finland and Norway approved the research plan and protocol as data was collected in both countries (Board of Ethics of Higher Education Institutions in Satakunta, Finland, 3.12.2016, and Regional Ethical Committee of Research, Sør-Øst D 2016/2242, Norway). A written informed consent was asked and obtained from all participants.

## Steve’s story: dynamics of *doing* masculinity

At the time of the interview, Steve worked as a technician and team leader in a middle-sized technology enterprise. On this note, four people were present during the interview, which took place at Steve’s home: Steve, the interviewer, Steve’s wife and a toddler-aged daughter. The wife tried to keep the toddler in another room while the interviewer and Steve discussed. However, every now and then the daughter, who wanted her father to play with her, interrupted the interview. Steve, however, was rather fluent in picking up his narrative trail after these interruptions.

In the beginning of the story, Steve draws a picture of “*normal childhood and adolescence”* that included family life, school and hobbies like those of his peers. He implies to “*a wild youth”* but continues to describe how the pain symptoms began and developed. Steve tells vividly how his years in vocational education and early years at work were fragmented by recurrent sick leaves, by examinations and tests one after another, and by being sent from one doctor to the next one. Steve describes his earlier background in fitness sports and weightlifting, which has given him an athletic body and big muscles. However, he had given up exercising due to persistent, agonizing musculoskeletal pains.

Not being accepted to military service due to his severe pains and poor functioning, seems to create a pivotal shift in Steve’s story; he does not fulfil the norms of men in his age group. To give background to the following two extracts, we need to explain that in Finland, men are obliged to join the army in the age of 20. However, prior to the military service, they must pass a medical examination, which is narrated in the following extract. The interviewer facilitates the storytelling with a simple question:

Steve “ … this [pain] is why they did not accept me to the army, you know … . [silence]”

Interviewer “Would you have liked to go?”

Steve “ … oh yes, many are like … umm … they don’t want to go there … but for me … it was a childhood dream! … I remember how the army doctor said: what do we do with a man who is not able to carry his backpack even for one kilometer? What do we do with a man who is not able to carry his assault rifle? Are you able to walk? … Well, yes but that’s just about it … yeah … You know, I have always kept myself fit … walk a lot and so on … [a lengthy example of earlier life style] … but now … I cannot do that anymore … I do not even consider, because you can feel it afterwards … like yesterday when there was new snow and it was fun to goof around in the snow with my daughter … but afterwards it was pure hell … pure hell, I say!”

In this passage, Steve zooms the discussion into a specific personal memory by using active voice (“I remember …”) and by changing his voice to show how the dialogue between him and the doctor evolved. He seems to ponder his identity by contrasting the desire to join the army and his current situation and points out that earlier he was fit to do physical activities, whereas now the pains and aches restrict substantially all his activities and daily life. This episode also reveals the silent, implicit expectations young men presumably encounter in society: You must be fit enough to be able to defend your country if necessary. Later in the story, Steve comes back to the topic about the army when he compares his functioning to that of his friend with another chronic musculoskeletal disorder

Steve “ … He is able to do physically demanding work … because he has proper medication … he is able to go to gym and lift weights … . he went to army … he was accepted there because he passed the tests … He passed the physical tests … [silence] … I don’t know if I would go there anymore, now that I have family and all … I guess I wouldn’t … ”

Interviewer “it was a blow to your ego, was it … ”

Steve “yes it was … it was bad for my ego … really bad … it was … it is hard … when you are not able to do ‘man-things’ anymore … that you were able to do earlier … ”

While Steve emphasizes the word “*He*” strongly in this episode, it is obvious that at the same time he is telling about his own life without saying it *de facto*:’ *He is able to do this and that -but I am not*’.

Moreover, the first sentence of the extract suggests that physically demanding work in Steve’s narrative account is “proper work for a man”. This norm becomes more evident as he portrays his current position as a team leader with a negative accent; it was impossible for him to continue in the physically demanding work, so he had to move to a job that was lighter. In the last sentence of the extract, the difference to his earlier life by referring to “man-things” is pointed out. Comparisons between himself and other men, and temporal comparisons between “now” and “then” are repeated several times in the story.

In the excerpt below, Steve tells about a lengthy period of searching for a diagnosis without success. He remarks:

“ … then finally, later that year, in occupational health care, they hit me with the label of fibromyalgia … and said that this is where they end, they are not going to do anything more … if you want something you have to go to the public sector … Well, I wanted to have a second opinion and went to public health care … after some examinations I was sent to a rheumatologist and she said ‘you have fibromyalgia’ … And I was like ‘I cannot have it … I am a man … I am a young man … ’ … you know, we have rheumatoid arthritis in our family, and I thought that that is what I have as well … I do not see this as a [real] disease even today, it is difficult to see it as one … and neither does the insurance system, they don’t see it as a disease either … ”

As we see it, Steve begins this part of the story with specific spatial indicators “*in occupational health care”* or *“in the public health care”* to show the roles these systems took in the process. A second marker for an important episode is Steve’s narrative strategy of recalling the dialogue between him and the doctor: *’if you want more, you have to go to the public sector’*. He does not only tell what was said but also imitates the way the doctors in question spoke and how he responded, by shifting his voice to display the different actors in the event. In this passage, Steve shows his moral indignation towards the fibromyalgia diagnosis for the first time clearly*: I can’t have it, I am a man!* He obviously has some knowledge about fibromyalgia being mainly diagnosed in women in their midlife as he questions whether the diagnosis can be correct for a man of his age. Later in the extract, he points out that he does not see fibromyalgia as a “real disease” and seeks for confirmation for this view by referring to insurance policies.

The encounters with health professionals, and especially with medical doctors, seem to have increased Steve’s distress and anxiety instead of supporting recovery or acceptance. When describing situations where he had felt that his symptoms were ignored or belittled by the doctors Steve uses language spiced with numerous swearwords to underline his frustration and anger. In the following extract, he also pinpoints the difference between meeting a male doctor or (female) nurse/public health nurse:

Steve “One [male] doctor said that I have this [fibromyalgia] because I am fat. I had already lost more than 10 kilos then. You are fat. Grannie’s illness. Overweight. Lose more … And he was a skinny kind of a guy, 170 cm tall and weighted like 40 kilos … I could have twisted him into a knot if I wanted to … And he comes and says that you are nothing but fat! Lazy bastard you are … Fuck, I could have lifted him on the wall! … But the nurses are different … they have this compassion in them … […] when they hear that you have this illness, they kind of put the professional role aside and the human person steps in … But when you get to see the male doctors … their attitude is different … much more negative … they belittle the symptoms, say that it’s all in the head and so on … It is so fucking infuriating!”

In this part of the story, Steve’s experiences on negotiations and tensions about the (male) body are clearly narrated. More specifically this comes to the fore when he characterizes the doctor as “a skinny type of guy” and thus, juxtaposes his weak bodily appearance with his own strong, muscular, yet painful body. As Steve has a background in weightlifting and other sports, the comments about overweight are perceived as derogatory, and seem to insult his perceived body image and masculinity deeply. Drawing on his own experiences, Steve generalizes the negative attitude towards fibromyalgia to all male doctors and expresses his positive experiences of female nurses as *“having this compassion in them”*.

At this point of the story, the deep tension between masculinity and fibromyalgia becomes very clearly demonstrated. Even more so as after receiving the diagnosis, Steve explores web-based support groups hoping to find other men with the same condition. However, as most of the participants were middle-aged women, Steve tells, he is not able to relate to their stories. Rather than that, he feels even lonelier and perceives himself as an outsider in the group. Several times during the story remarks or metaphors about fibromyalgia as an “*old grannies” disease’* or *“for women only”* are made. Moreover, because earlier physically strenuous hobbies due to the pains and aches had to be abandoned some social relationships were also lost. These feelings of not-belonging and otherness are expressed, e.g., through recurrent reflections like *“I am not one of the guys anymore”* or *“what friends? … I don’t have [male] friends anymore”.*

However, there are nuances to the hegemonic masculinity that the story seems to reflect. During his telling, Steve adds in that because of having a family, going to the army would not be as meaningful for him now as it was earlier. This hesitation might suggest a strong gender equality orientation, typical for young men in the Nordic countries. Later Steve phrases the importance of family and caring more clearly, when he tells about side effects of the medication he had earlier for the symptoms of fibromyalgia.

Steve ” … I had a low dose [antidepressive medication] … but the effect on me was too strong … it led to lack of emotional life … it took everything away … lust, desire, warm thoughts, happy thoughts … I did not have even cold thoughts anymore … I didn’t even get angry about anything … l was like … you know … I guess that is how lobotomy patients would feel … . […] … it does not fit in with your work … you have to care about things at work … you have to care about things at home, you have to take care of your daughter and wife … care about the home … .but when you have that medication you are like … like you were sitting in the front yard and watching your house burn down … you just couldn’t care less … “

In this rather intense episode, Steve put emphasis on that he does not want to be “*emotionless lobotomy-patient*” but a father and a husband who takes care of the family, home and work, and who is able to show his compassion and affection as well as other feelings. It is also noteworthy, that in this episode, Steve uses passive voice (you have to …) and thus zooms out from his own personal experience to claim a more general perspective of how a man *should be*, behave and act in our society.

The family theme continues later as the toddler-aged girl rushes in to the room once again to show her toys and insists her father to play with her. Steve persuades her gently to return to the living room with her Mom and promises to play with her later. The reflection on father–daughter relation after the interruption is prompted by interviewer’s question:

Interviewer: “Does the illness or symptoms you have affect your relationship with your daughter?”

Steve “Yes, yes it does … to playing with her outdoors, for instance … .lifting her up and down [to a swing] … it takes its toll … but when you are not able to lift, when you simply don’t have the strength … even if you want to … and then she would be like ‘why not, dad?’ … and I would really like to do that … but try to explain to a toddler that because it hurts … because dad is in pain … try to explain to a 2- year-old that dad is not able to pull you in the sled because it hurts! … so, yes it has an impact … especially on playing with her … and when you have more pain, you tend to be grumpier … more bad-tempered … “

In this passage, Steve’s frustration of not being able to be the “ideal father” or “the father-body” he wants to be is clearly audible. This frustration might reflect contemporary Nordic societies where family-patterns are distinctly changing, and in which men due to gender equality policies take more responsibility on raising the children and sharing the domestic chores in comparison to earlier generations (see Solbrække, 2016). This change or enlargement of traditional masculinity might shed light on why Steve seems to be struggling to find ways of being a modern husband and father. Not only does his bodily situation restrict him from traditionally manly subject positions such as being active in military service or being a credible patient, but also denies him from being the active and protective father he aims for.

Overall, Steve’s life seems to turn out different from what he had expected. The following extract is from the last part of the story, where he ponders what to do with his life in the future and where very few positions as an active and independent man seems to be reachable:

“ … The car hobby had to be left behind … I barely manage to change the tires nowadays and that’s all … You do not crawl under the car anymore … or putter around in the garage hall and fix cars … all that I had to give up. And you are not able go to the gym either … it is so fucking irritating that you cannot do anything but eat and get fat … it’s about having nothing to do … I’m not able to do any forest work or things like that anymore … or anything that I used to enjoy doing … […] … I want to get back to doing things myself … without always asking others to help … without thinking all the time how to avoid pain … I wish I could have another diagnosis and to get proper medication and treatment … to be able to do things again … ”

In this passage, Steve lists his earlier hobbies that can all be perceived as “traditionally masculine”: fixing cars, lifting weights and being occupied with forest work. Thus, it seems that fibromyalgia, and especially the pains and aches, limit not only his physical and social functioning but also “*doing*” the masculine identity through leisure time activities. The expressed wish of getting another diagnosis and proper medication implies that Steve anticipates no improvement in his functioning in the near future.

## Discussion

In the beginning of this research project, we were interested in men’s experiences of living with fibromyalgia; what kind of symptoms did the men have and how did the illness influence their daily life? However, as we were following a narrative approach and asking for life stories, we soon discovered that being a man or retaining one’s idea of masculine identity were repeatedly intertwined with the general storylines of the illness stories. Therefore, we found it interesting and important to explore the data in detail using a gender approach as an analytical lens. We wanted to elucidate how men with fibromyalgia “*do*” and negotiate their gender in their told life stories and how do they reflect on the cultural norms of masculinity.

Before discussing the results of the study more broadly, it is necessary to address some methodological considerations. Firstly, the findings of this study may be limited due to the sampling methods that were used. Although the study raised lively discussions in on-line support groups, we failed to reach large amounts of male patients with fibromyalgia. This may be a limitation of the study. However, we managed to reach participants with different educational, professional and social backgrounds who were willing and eager to share their experiences about living with fibromyalgia. Moreover, referring to Malterud, Siersma, and Guassora (2016), we believe that sufficient “information power” of qualitative data can be reached through specificity of the sample, in-depth interviews and rigorous analysis rather than through a more superficial analysis of larger number of interviews.

Secondly, using one story as an example per excellence also needs to be clearly justified. As all life stories are unique and personal, they are to be appreciated for their own merit. However, despite the differences in individual experiences, some commonalities can be drawn across the data that may improve our understanding about the gendered experiences of fibromyalgia. In our study, the men particularly explored the changes in their masculine identity by comparisons to other men, by comparisons to time before the onset of the symptoms and now, and by discussing the silent and explicated expectations and beliefs of how men should behave and act in our contemporary societies. As the dynamics of “*doing gender*” demonstrated here through Steve’s story very distinctly also runs through the life stories of the other men in this study, we believe our findings to be relevant for the whole sample. Furthermore, as pointed out by Riessman (2003) and Mattingly and Lawlor (Mattingly & Lawlor, 2000), individual cases matter. That is, by exploring and comparing the smallest details of narrative accounts it is possible to reveal the contrasting meanings and interpretive complexity of individual experiences. In this study, we reached for a deeper understanding of “*doing gender”* through analysing also the linguistic and performative aspects of the narratives we heard.

Finally, we must bear in mind that both interviewers in this study are women. This may have had impact on how the men talked about their masculinity. Moreover, it is likely that the participants’ background factors such as age and vocation, have an impact on the men’s views on the illness as such, and on masculinity. Most of our participants worked in male-dominated workplaces which may have influenced their views of the societal expectations and men’s role in family from a rather traditional perspective as suggested by earlier studies (Branney & White, 2008; O’Brien et al., 2005; Samulowitz et al., 2018). O’Brien et al. (O’Brien et al., 2007) remarked that men with different illnesses believed that they were expected to remain “strong and silent” regarding their health problems, especially when related to anxiety and depression. However, the fact that the men in our study disclosed also sensitive issues and difficult life events in their narratives, suggests that the interviewers were perceived as trustworthy and easy to talk to. We have included authentic extracts from the data in the result section to help the readers to evaluate the accuracy and relevance of our interpretations.

In the data, masculinity was discussed in relation to onset and deterioration of the symptoms, for example, when the men noticed that they were unable to manage their work or hobbies. The men also pondered their masculinity in association with encounters with health professionals during the diagnostic process, and with changes in their lifestyle, they had experienced after the diagnosis – willingly or unwillingly. Being diagnosed with fibromyalgia caused mixed feelings in many of the participants. Although it was a relief to have a name for the pains and aches and other symptoms, it raised questions about identity in general and about masculinity in particular. Changes in their bodily performance forced the men to re-negotiate and re-construct their masculinity. The transition from experiencing a strong, active and reliable body to experiencing a painful, vulnerable and helpless body was perceived as difficult and fundamental. This finding is in line with the statements about the vulnerability of traditional hegemonic masculinity, especially when it is represented through bodily performances (Connell & Messerschmidt, 2005; Nolan, 2013; O’Brien et al., 2007). Nolan’s review (Nolan, 2013) about men with spinal cord injuries, underlined that the sense of loss of masculine identity is powerful, and may result in preoccupation of previous levels of functioning and in constant yearning of what was of what might have been.

The leisure time activities seem to offer a social environment where men can spend time with other men and experience a feeling of “belonging” to a group, but which by developing fibromyalgia becomes a lost landscape. For example, when Steve had to give up his hobbies because of the pains, he also gave up part of his social life and reference groups. This kind of changes in social relationships and leisure time activities due to fibromyalgia were common throughout the stories we were told, albeit the extent of the changes varied in-between the individuals. This pattern resonates with Emslie et al. (2006) who pointed out that “being one of the boys” in social environments seems to be important to men’s gender identity. Experiences of otherness or not belonging to a group like those presented in our findings have also been found in earlier studies about fibromyalgia (Paulson et al., 2002) as well as in other long-term illnesses (Emslie et al., 2006; Flurey et al., 2017; O’Brien et al., 2005). Flurey et al. (2017) noticed in their study among men with rheumatoid arthritis, that ability to maintain or retain physical activities or sport-related hobbies helped the men to avoid the feeling of “otherness” among men, which in turn supported their masculine identity. Moreover, in many chronic illnesses and disabilities it might be easy to find a “man club”—a peer group of other men with same diagnosis, but in fibromyalgia, this seems to be difficult, if not impossible, due to the small number of male patients.

In earlier studies concerning fibromyalgia, the encounters with healthcare professionals have been described as challenging or filled with negative attitudes and disbelief from the health professionals’ side (Conrad & Barker, 2010; Katz et al., 2010; Paulson et al., 2002). McMahon, Murray, Sanderson, and Daiches (2012) underlined that encounters with health professionals, in which the patients are not heard or believed, may prolong the adjustment process, hinder the access to appropriate treatment, and even threaten the identity of the patient. This was apparent also in our study. In the episode told by Steve’s in which the “skinny” doctor heavily disapproves his bodily appearance, the disconfirming and negative attitudes became clearly audible: *there is nothing wrong with you, you are just fat!* Not only does this kind of a remark belittle the pains and aches the patient experiences, but also insults deeply the very core of the patient’s identity and integrity. Interestingly, similar experiences of humiliation and disbelief have been described in studies about women with chronic widespread pains. The women in these studies portrayed a vulnerable position in relation to encounters with health professionals (Werner & Malterud, 2005, 2003). According to our results, men with fibromyalgia seem to be equally vulnerable in terms of gender identity and dignity in these encounters.

Our results suggest that men with fibromyalgia seem to be lost in transition from traditional hegemonic masculinity to more nuanced “*alternative masculinities”* (see also Solbraekke, 2016). However, they seemed also to resist “*marginalized masculinity*” by not wanting to be seen as disabled. Steve’s story illustrates clearly how telling about his life with fibromyalgia involves a constant re-negotiation of his masculinity (or masculinities). More particularly, he seemed to be balancing between the previous-Me and the unwanted-Me (strong manly man vs. man with a disability). In addition, he realized that it might be impossible to retain the masculine identity he had earlier, or to reach his own ideal masculinity. At the same time, Steve seemed to resist the traditional hegemonic masculinity by expressing a warm, caring attitude towards his closest ones. He seemed to be struggling to construct a *possible masculinity* in his current situation. Similarly, Abbott, Jepson, and Hastie (2016) noticed that young men with muscle dystrophy struggled to find their own way of defining their masculine identity; the men discussed their similarities and differences from stereotypes of “ordinary”, non-disabled men and underlined features like maturity, insights and wisdom as the very core of “being a man” (Abbott et al., 2016).

According to earlier scholars (Hydén, 1997; Mattingly & Lawlor, 2000; Riessman, 2003) by telling illness narratives, people strive to find coherence and continuity to their life course that is disrupted by the illness. In terms of narrative theories (Davies, 2002; Hydén, 1997), Steve’s story does not seem to have a clear end or a resolution that would give a meaning to earlier events and experiences; rather, the question of gender identity remains unanswered.

## Conclusions

Our results suggest that fibromyalgia shapes the way men see themselves as men. The interplay between illness and gender seems to lead to re-constructing their masculinity in terms of what is possible. The dilemma that was implied albeit not necessarily clearly explicated in the stories is: “*I cannot be the man I used to be or want to be, and I do not know what kind of a man I can be with this illness”*. Therefore, self-management and rehabilitation of fibromyalgia is not only about learning to manage the symptoms, it is also about the struggle to find continuity and coherence in life through re-constructing gender identity that is acceptable both for the individual himself and for the community.
